# Sjögren’s syndrome with and without neurological involvement

**DOI:** 10.1007/s00415-023-11613-5

**Published:** 2023-02-18

**Authors:** Tabea Seeliger, Emelie Kramer, Franz Felix Konen, Nadine Zehrfeld, Sonja Beider, Nils Kristian Prenzler, Vega Gödecke, Torsten Witte, Thomas Skripuletz, Diana Ernst

**Affiliations:** 1 Department of Neurology, Hannover Medical School, Carl-Neuberg-Str. 1, 30625 Hannover, Germany; 2 Department of Rheumatology and Immunology, Hannover Medical School, Hannover, Germany; 3 Department of Otolaryngology, Hannover Medical School, Hannover, Germany; 4 Center for Rare Diseases, Hannover Medical School, Hannover, Germany

**Keywords:** Primary Sjögren’s syndrome, Sjögren-associated neuropathy, Neuro-Sjögren, Neurological manifestations of Sjögren’s syndrome

## Abstract

**Objective:**

Neurological manifestations of Sjögren’s syndrome can be severe but also treatment-responsive. We aimed to systematically evaluate neurological manifestations of primary Sjögren’s syndrome and find clinical features allowing sufficient identification of affected patients (pSSN) among those with Sjögren’s syndrome without neurological involvement (pSS).

**Methods:**

Para-/clinical features of patients with primary Sjögren’s syndrome (2016 ACR/EULAR classification criteria) were compared between pSSN and pSS. At our university-based center, patients with suggestive neurological symptoms undergo screening for Sjögren’s syndrome, and newly diagnosed pSS patients are thoroughly evaluated for neurologic involvement. pSSN disease activity was rated by the Neurological Involvement of Sjögren’s Syndrome Disease Activity Score (NISSDAI).

**Results:**

512 patients treated for pSS/pSSN at our site between 04/2018 and 07/2022 were included (238 pSSN patients [46%] vs. 274 pSS patients [54%], cross-sectional design). Independent predictors of neurological involvement in Sjögren’s syndrome were male sex [*p* < 0.001], older age at disease onset [*p* < 0.0001], hospitalization at first presentation [*p* < 0.001], lower IgG levels [*p* = 0.04] and higher eosinophil values (treatment-naïve) [*p* = 0.02]. Univariate regression additionally showed older age at diagnosis [*p* < 0.001], lower prevalence of rheumatoid factor [*p* = 0.001], SSA(Ro)/SSB(La) antibodies [*p* = 0.03; *p* < 0.001], higher white blood cell count [*p* = 0.02] and CK levels [*p* = 0.02] (treatment-naïve) in pSSN.

**Interpretation:**

Patients with pSSN had different clinical characteristics than patients with pSS and represented a large proportion of the cohort. Our data suggest that neurological involvement in Sjögren’s syndrome has been underestimated. Intensified screening for neurologic involvement should be included in the diagnostic algorithm for Sjögren’s syndrome, especially in males of older age and with severe disease course requiring hospitalization.

## Introduction

Sjögren’s syndrome (pSS) is characterized by an autoimmune lymphocytic infiltration of the salivary and lachrymal glands, causing sicca symptoms of mucosal structures often accompanied by extra-glandular manifestations. The latter can involve the lungs, liver, kidneys, the hematological and the nervous system and can lead to severe organ damage. In particular, the neurological manifestations of Sjögren’s syndrome have been the focus of recent research leading to a better understanding of the neurological features [1–5] and highlighting the associated limitation of quality of life [6]. Interestingly, it has also been shown that the disease course of affected patients may be more severe than previously anticipated [7, 8], but responds well to immunosuppressive treatment [9, 10]. In several previous studies, there was evidence that patients with Sjögren’s syndrome and neurologic involvement present with distinct clinical features compared with patients with Sjögren’s syndrome without neurologic involvement: For example, patients with Sjögren’s syndrome with overt neurological manifestations appear to have a more balanced female to male ratio [8, 11–13]. Nevertheless, the analyzed cohorts show great variability in terms of size, definition of neurological involvement, applied classification criteria for Sjögren’s syndrome, and inclusion of patients with primary and/or secondary Sjögren’s syndrome [14–16]. Hence, systematic workup of patients with additional neurological involvement in Sjögren’s syndrome is still insufficient for early identification and thus for early adequate treatment initiation. Therefore, our aim was to systematically assess the neurological manifestations of Sjögren’s syndrome at our specialized university hospital in Germany and to find clinical features that allow sufficient identification of affected patients among those with Sjögren’s syndrome.

## Methods

### Study design

Patients treated for pSS between 04/2018 and 07/2022 in the Department of Neurology, the Department of Rheumatology and Immunology, and the Center for Rare Diseases at Hannover Medical School were included in this study. Data on clinical and para-clinical features of patients with Sjögren’s syndrome were retrospectively collected and analyzed for differences between patients with (pSSN) and without additional neurological involvement (pSS). Laboratory findings were recorded from available time points before immuno-modulative treatment. Information on the type of initial presentation to the outpatient clinic or via hospital admission was also recorded. Parts of the analyzed cohort have been included in previously published studies with focus on other clinical and/or para-clinical aspects [2, 4, 7, 8, 17–22]. The study was approved by the local ethics committee (8172_BO_K_2018; 8179_BO_S_2018) and was conducted in accordance with the declaration of Helsinki. It was not appropriate or possible to involve patients or the public in the design, conduct, reporting, or dissemination plans of our research.

### Diagnosis of Sjögren’s syndrome

The diagnosis of Sjögren’s syndrome was reappraised for each patient and had to meet the current classification criteria for Sjögren’s syndrome [23]. Patients with suspected secondary Sjögren’s syndrome and overlap with IgG4-associated disease were not included in this analysis.

### Disease activity scoring and assessment of neurological involvement

Disease activity in Sjögren’s syndrome was quantified using the established EULAR Sjögren’s Syndrome Disease Activity Index (ESSDAI) [24] at the time of maximum disease activity. The ESSDAI consists of 12 sub-scores for constitutional, lymphadenopathic, glandular, articular, cutaneous, pulmonary, renal, muscular, peripheral and central nervous systems, and hematologic and biologic involvement, which are summarized into a total score. It ranges from 0 (no systemic involvement) to 123 (maximal systemic involvement) and is the established scoring tool for disease activity in Sjögren’s syndrome.

Subjective symptom severity was assessed using the EULAR Sjögren’s Syndrome Patient Reported Index (ESSPRI), which is implemented into our clinical routine [24]. This score depicts the severity of symptoms from the patient’s perspective and is calculated as the mean of three sub-scores: Dryness, Fatigue and Pain—each on a scale from 0 (no complaints) to 10 (maximum discomfort imaginable).

Neurological involvement was assumed only for legitimate symptoms and in accordance with the neurologic sub-scores of the ESSDAI: Peripheral nervous system [PNS] (defined as peripheral neuropathy with presumed autoimmune background, cranial and/or phrenic nerve affection); central nervous system [CNS] (defined as cerebral vasculitis, vascular lesions with presumed autoimmune background, encephalitis, abacterial meningitis, myelitis) and/or muscular system (defined as biopsy-proven myositis or myositis apparent on image morphology [i.e., MRI with contract medium or positron emission tomography] combined with elevated CK values). This comparatively strict separation was deliberately implemented to avoid misinterpretation of non-specific neurological symptoms as neurological involvement of Sjögren’s syndrome. The only exception in this procedure was allowed for patients with biopsy-proven Small-Fiber Neuropathy diagnosed according to the current diagnostic criteria [25]—an entity that had not been included in the original ESSDAI score. In this study, these patients were scored with a low activity level of PNS involvement [5 points] because their pure sensory polyneuropathy was histopathologically detectable. In accordance with the suggestions of the ESSDAI, chronic pain syndromes, fatigue, myasthenia gravis, and stroke or transient ischemic attacks of known non-immunologic origin did not lead to the diagnosis of pSSN. The additional diagnosis of multiple sclerosis, isolated evidence of cognitive impairment, and neuromyelitis optica spectrum disorder also did not lead to the diagnosis of neurological involvement in Sjögren’s syndrome. However, these symptoms were recorded and additionally analyzed for included patients.

During the preparation of the manuscript, it became apparent that the ESSDAI-derived neurologic scores for PNS, CNS, and the muscular system provided a rough estimate of disease activity in neurologic involvement of Sjögren’s syndrome. However, several major problems emerged during the in-depth study of the included patients with neurologic involvement: First, the assessment of cranial nerve impairment is proposed to be divided by central and peripheral origin, which may be challenging in clinical practice. Second, a combination of multiple disorders with the same level of activity is only scored once, resulting in a narrower range of possible outcomes. Finally, in addition to the omitted patients with small fiber neuropathy, patients with myasthenic syndrome (reduced motor performance on repetitive tasks, double vision, ptosis with symptoms progressing over the course of the day, often in combination with myalgia and autoimmune antibodies) and phrenic nerve affection are not scored, although all three entities result in a significant reduction in patient’s quality of life.

Therefore, the Neurological Involvement of Sjögren’s Syndrome Disease Activity Index (NISSDAI) was developed to adequately assess the disease severity with a pure focus on the neurological manifestations of Sjögren’s syndrome (Table 1). The score was evaluated only for patients with confirmed neurologic involvement via neurologic ESSDAI sub-scores. It additionally considers small fiber neuropathy, myasthenic syndrome, and vascular lesions with presumed autoimmune background as they severely affect patients’ daily life, even if they are not considered specific neurological symptoms of Sjögren’s syndrome.

**Table 1:** Neurological involvement of Sjögren’s syndrome disease activity score (NISSDAI)

Neurological symptoms	Points
Peripheral sensory neuropathy (on NCS or biopsy-proven Small-Fiber Neuropathy)	5
Peripheral motor neuropathy (on NCS with reduced motor performance)	10
Myositis evident on biopsy or image morphology combined with CK elevation	10
Myelitis and/or encephalitis and/or abacterial meningitis	10
Cerebral vasculitis/vascular lesions of presumed autoimmune background	10
Cranial nerve impairment or phrenic nerve affection	5
Myasthenic syndrome	5
Proven cognitive impairment on confrontational testing	5

The total score represents a sum score of all apparent neurologic features and ranges from 0 to 60. The NISSDAI was only scored for patients with evident neurological involvement

### Statistical and graphical analysis

Binary variables were statistically analyzed for group differences using chi^2^ test. Metric variables were visually analyzed for normal distribution because of the large cohort size. Group differences for parametric variables were analyzed using two-sided t test, whereas non-parametric data were processed using Wilcoxon test. Subsequently, univariate logistic regression analysis was performed for all significant variables. This first part of statistical analysis was performed using STATA® 16.1 Texas, USA. Furthermore, logistic regression analysis was performed using R environment for statistical computing version 4.0.4 (R Foundation for Statistical Computing, Vienna, Austria). Using only available data, uni-variable logistic regression analysis was performed on candidate variables using the “lme4” package. For multivariable testing, missing values of potential variables were imputed by multivariable imputation using the “mice” package with settings to 5 multiple imputations with 50 iterations. Algorithm convergence was visually ascertained. The pooled data were then used for multivariable modeling. Variables were added to the model if they were found to be contributory by testing against a “null-model” via sequential likelihood ratio test. Final pooled estimates are provided as odds ratios with 95% confidence interval and p values. The significance level was set at 0.05. Graphs were created by GraphPad Prism Version 9.4.0 (673) ©1992–2022 GraphPad Software, LLC.

## Results

512 patients with primary Sjögren’s syndrome according to the current ACR/EULAR classification criteria [23] from across Germany were included in this study. 238 of the included patients (46%) suffered from additional neurological involvement according to the proposed ESSDAI sub-scores for muscular, and peripheral and central nervous system involvement and were subsequently assigned to the pSSN subgroup. The remaining 274 patients (54%) with Sjögren’s syndrome but without the corresponding neurologic features were assigned to the pSS subgroup. The ratio of females to males was more balanced in patients with pSSN (65% female) than in patients with pSS (female 85%, *p* = 0.001). Analysis of other baseline characteristics of patients with pSSN showed significantly higher age at onset (*p* < 0.0001) and at diagnosis (*p* < 0.0001), more frequent initial presentation via hospitalization (*p* < 0.001), and significantly less frequent SSA(Ro) antibody positivity (*p* = 0.03) than patients with pSS without neurologic involvement. The ESSDAI reached significantly higher total scores in patients with pSSN (mean 18.2 ± 21) than in patients with pure pSS (mean 7.7 ± 7.3; *p* < 0.0001). The full analysis is shown in detail in Table 2.

**Table 2:** Baseline characteristics for patients with primary Sjögren’s syndrome compared for the subgroups with neurological involvement (pSSN) and without (pSS)

	pSSN	pSS	*p* value
*N* total	512		n/a
*N* (%)	238 (46%)	274 (54%)	n/a
Female, *N* (%)	154 (65%)	233 (85%)	**0.001**
Age at onset, median (IQR) [y]	55 (43.5–64.5)	45 (33–56)	** < 0.0001**
Age at diagnosis, median (IQR) [y]	59.5 (50–68.5)	51 (39.5–60)	** < 0.0001**
Initial presentation mode at the site via hospital admission, *N* (%)	127 (58%)	71 (31%)	** < 0.001**

ACR/EULAR classification criteria 2017	Reported assessment	Pathological, *N* (%)	Reported assessment	Pathological, *N* (%)	
Evident xerophthalmia [by Schirmer’s test]	235	193 (82%)	273	214 (78%)	0.01
Evident xerostomia [by Saxon test]	229	114 (50%)	259	130 (50%)	0.9
SSA(Ro) antibody positivity	235	125 (53%)	267	167 (63%)	**0.03**
Sialadenitis grade ≥ 3 (Chisholm and Mason)	173	144 (83%)	149	134 (90%)	0.08
2016 Focus score, mean ± sd	4.7 ± 1.1		4.6 ± 1		0.2
Disease activity					
ESSDAI, mean ± sd	18.2 ± 21		7.7 ± 7.3		** < 0.0001**
ESSPRI total, mean ± sd	5 ± 2.4		4.8 ± 2.3		0.5
ESSPRI subscore: dryness, median (IQR)	5 (2–7)		5 (2–7)		0.5
ESSPRI subscore: fatigue, median (IQR)	6 (3–8)		6 (3–8)		0.6
ESSPRI subscore: pain, median (IQR)	5 (3–8)		4 (1–7)		0.1
NISSDAI, mean ± sd	10.4 ± 4.9		n/a		n/a

*N* number, *y* years, *IQR* interquartile range, *n/a* not applicable, *sd* standard deviation, *NISSDAI* Neurological Involvement of Sjögren’s Syndrome Disease Activity Index, bold values refers statisitcally significant finding

### Neurological features in Sjögren’s syndrome

Analysis of neurological features in all 512 included patients with primary Sjögren’s syndrome revealed that peripheral nervous system (PNS) involvement was most common: 167 patients had peripheral neuropathy as revealed by nerve conduction studies (NCS). The latter showed axonal neuropathy in 34 patients (20%), demyelinating neuropathy in 77 patients (46%), and mixed axonal and demyelinating neuropathy in 52 patients (31%), whereas there were no electro-diagnostic reports for 4 patients (2%). Furthermore, there were 30 additional patients with small fiber neuropathy (according to the current diagnostic criteria [25] without abnormalities in the NCS), raising the total number of patients with PNS involvement to 197 (38%). 19 patients suffered from myositis (4%), which was diagnosed based on histopathologically suggestive findings on muscle biopsy in 18 patients and based on suggestive enhancement on positron emission tomography combined with CK elevation in one patient.

**Fig. 1:**
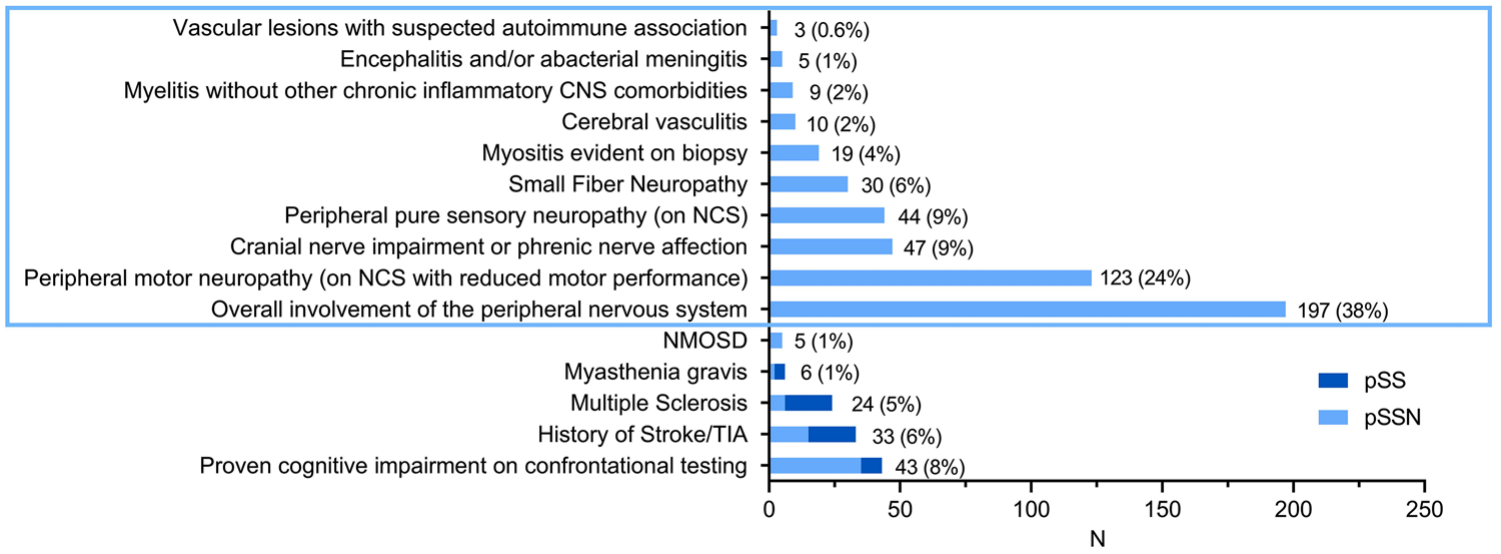
Frequency of neurological features in all 512 included patients, with blue rectangle indicating features that led to the allocation to the subgroup of Sjögren’s syndrome with neurological involvement. *pSS* primary Sjögren’s syndrome, *pSSN* primary Sjögren’s syndrome with neurological involvement, *NCS* nerve conduction studies, *TIA* Transient Ischemic Attack, *NMOSD* Neuromyelitis Optica Spectrum Disease

In the entire cohort, there was a notable accumulation of patients with multiple sclerosis (*N* = 24, 5%), and NMOSD (*N* = 5, 1%) and myasthenia gravis diagnosed by electro-diagnostic abnormalities on repetitive nerve stimulation and/or detectable myasthenia-associated antibodies (*N* = 6, 1%). The latter was diagnosed based on findings at the repetitive nerve stimulation in 2/6 patients and based on antibody positivity in 4/6 patients. In addition to the described 6 patients with diagnosed myasthenia gravis, there were 3 more patients displaying the clinical picture of a treatment responsive myasthenic syndrome (reduced motor performance on repetitive tasks, double vision, ptosis with symptoms progressing over the course of the day, myalgia) but without evidence of unambiguous findings for autoantibodies or on repetitive nerve stimulation. The complete workup is shown in Fig. 1.

Analysis of the 238 SSN patients revealed that 188 patients (79%) had exclusively one of the pSSN-defining neurological features (blue rectangle in Fig. 1). Another 49 patients (21%) were diagnosed with two, and another single patient (0.4%) with three of the mentioned neurological disorders associated with Sjögren’s syndrome.

### Comorbidities, immunological parameters, and baseline laboratory findings

Analysis of comorbidities showed no significant group differences for cardiovascular risk factors. Allergies were reported less frequently in the pSSN group (*p* = 0.04), especially when comparing drug-related allergies (*p* = 0.04) and seasonal allergies (*p* = 0.04). The full data are displayed in Table 3.

**Table 3:** Comorbidities concerning cardiovascular risk factors and reported allergies in patients with primary Sjögren’s syndrome with neurological involvement (pSSN) and without (pSS)

	pSSN		pSS		*p* value
	Information available	Pathological, *N* (%)	Information available	Pathological, *N* (%)	
Cardiovascular risk factors					
History of TIA	211	2 (1%)	261	8 (3%)	0.1
History of stroke	224	13 (6%)	271	10 (4%)	0.3
KHK	223	11 (5%)	270	11 (4%)	0.6
History of smoking	149	78 (52%)	239	107 (45%)	0.1
Diabetes mellitus	221	21 (10%)	272	14 (5%)	0.07
Reported allergies					
Allergy affected patients	221	84 (38%)	271	128 (47%)	**0.04**
Drug-related	221	43 (19%)	271	74 (27%)	**0.04**
Other (nutritional, contact dermatitis, insects, bugs)	221	62 (28%)	271	96 (35%)	0.1
Seasonal	221	31 (14%)	271	57 (21%)	**0.04**

*N* number, *TIA* transient ischemic attack, bold values refers statisitcally significant finding

Comparison of laboratory parameters revealed a significantly lower prevalence of rheumatoid factor (*p* = 0.001) and SSB(La) antibodies (*p* < 0.001) in patients with pSSN than in patients with pSS. Furthermore, laboratory findings at baseline and before treatment with immunosuppressants showed significantly lower values for IgG (*p* = 0.02) and higher values for white blood cell counts (WBC, *p* = 0.004), eosinophils (absolute: *p* < 0.0001; relative: *p* < 0.0001) and CK (*p* = 0.01) in the pSSN group when compared to the pSS group. All laboratory features are listed in Table 4.

**Table 4:** Laboratory findings for immunological parameters (maximal pathological aberration) and baseline parameters (before therapy initiation) for patients with primary Sjögren’s syndrome with (pSSN) and without neurological involvement (pSS)

	pSSN		pSS		*p* value
	Available	Pathological, *N* (%)	Available	Pathological, *N* (%)	
Immunological parameters					
ANA	218	116 (53%)	252	152 (60%)	0.1
Rheumatoid factor	232	40 (17%)	263	78 (30%)	**0.001**
Alphafodrin antibody (IgA)	204	69 (34%)	250	88 (35%)	0.8
SSB(La) antibody	234	26 (11%)	260	69 (27%)	** < 0.001**
Sm antibody	214	3 (1%)	224	6 (3%)	0.4
U1SnRNP antibody	225	4 (2%)	225	9 (4%)	0.3
Scl 70 antibody	214	0 (0%)	224	1 (0.5%)	1.0
CENPB antibody	215	4 (2%)	223	9 (4%)	0.3
Jo1 antibody	217	0 (0%)	231	2 (1%)	0.2
CCP antibody	63	4 (6%)	146	3 (2%)	0.2
Baseline parameters before therapy initiation					
IgG, mean ± sd [g/l]	108	11.8 ± 4.1	137	13.3 ± 5.4	**0.02**
IgA, median (IQR) [g/l]	102	1.9 (1.3–3)	133	2.3 (1.6–3)	0.07
IgM, median (IQR) [g/l]	99	1.1 (0.7–1.5)	134	1.2 (0.8–1.6)	0.3
WBC, median (IQR) [thousand/µl]	142	6.3 (5.3–7.5)	160	5.7 (4.6–7.2)	**0.004**
RBC, mean ± sd [million cells/µl]	142	4.6 ± 0.5	159	4.6 ± 0.4	0.9
Hemoglobin, mean ± sd [g/dl]	142	13.7 ± 1.4	160	13.5 ± 1.2	0.1
Hematocrit, median (IQR) [%]	141	40.6 (38.1–43.1)	160	40.1 (37.6–41.6)	0.09
Platelets, mean ± sd [thousand/µl]	141	251 ± 70	159	257 ± 65	0.5
Neutrophils abs, median (IQR) [thousand cells/µl]	131	3.7 (3–4.6)	152	3.3 (2.7–4.3)	**0.03**
Neutrophils, mean ± sd [%]	131	59.5 ± 10.8	152	59.3 ± 11.1	0.9
Eosinophils abs, median (IQR) [cells/µl]	130	0.2 (0.1–0.2)	152	0.1 (0.04–0.2)	** < 0.0001**
Eosinophils, median (IQR) [%]	133	2.2 (1.5–3.8)	154	1.7 (0.8–2.7)	** < 0.0001**
Basophils abs, median (IQR) [cells/µl]	131	0.01 (0–0.05)	154	0.03 (0–0.06)	0.07
Basophils, median (IQR) [%]	131	0.6 (0.4–0.7)	155	0.5 (0.4–0.9)	0.7
Monocytes abs, mean ± sd [cells/µl]	132	0.6 ± 0.62	154	0.51 ± 0.6	0.2
Monocytes, mean ± sd [%]	130	8.5 ± 2.5	154	8.1 ± 2.6	0.2
Lymphocytes abs, mean ± sd [cells/µl]	132	2.3 ± 4.7	154	1.7 ± 0.6	0.09
Lymphocytes, mean ± sd [%]	130	28.4 ± 9.9	154	29.4 ± 9.4	0.4
CRP, median (IQR) [mg/l]	136	1.4 (0.8–4.1)	156	1.3 (0.7–3.1)	0.4
eGFR, median (IQR) [90 ml/min/1.73m^2^]	128	92 (74.5–100)	149	89 (76–100)	0.7
CK, mean ± sd [U/l]	136	147 ± 156	149	105 ± 115	**0.01**
AST, median (IQR) [U/l]	132	25 (21–34)	140	24 (20–29)	0.1
ALT, mean ± sd [U/l]	120	32 ± 44	158	28 ± 32	0.4
YGT, mean ± sd [U/l]	125	33 ± 58	155	28 ± 26	0.4

*pSS* primary Sjögren’s Syndrome, *pSSN* primary Sjögren’s Syndrome with neurological involvement, *N* number, *ANA* antinuclear antibodies, *IgG* immunoglobulin G, *IgA* immunoglobulin A, *IgM* immunoglobulin M, *WBC* white blood cells, *RBC* red blood cells, *sd* standard deviation, *abs* absolute, bold values refers statisitcally significant finding

### Logistic regression analysis

Univariate logistic regression analysis confirmed statistically significant effects for all variables with previously established significant group differences—except for absolute Neutrophils (*p* = 0.1) and seasonal allergies (*p* = 0.05). Only the variables sex, age at onset, type of initial presentation at the site via hospital admission, and absolute values for eosinophils and IgG improved the multivariate statistical model. Independent predictors of neurological involvement in Sjögren’s syndrome were male sex (OR (calculated for female sex) 0.33 [0.16–0.65 95% CI], *p* = 0.001), older age at onset (OR 1.004 [1.002–1.006 95% CI], *p* < 0.0001), hospital admission at first presentation (OR 3.2 [1.71–5.99] 95% CI], *p* < 0.001), as well as lower absolute values for IgG (OR 0.92 [0.86–0.998 95% CI], *p* = 0.04) and higher values for eosinophils (OR 1.33 [1.05–1.68 95% CI], *p* = 0.02). The full analysis is shown in Table 5.

**Table 5:** Uni- and multivariate regression analyses for variables that previously showed significant group differences between patients with Sjögren’s syndrome and neurological involvement and those without

Variable	Univariate OR [95% CI]	Univariate *p* value	Multivariate OR [95% CI]	Multivariate *p* value
Female sex	0.32 [0.21–0.49]	** < 0.001**	0.33 [0.16–0.65]	**0.001**
Age at onset	1.04 [1.02–1.05]	** < 0.001**	1.004 [1.002–1.006]	** < 0.0001**
Age at diagnosis	1.04 [1.03–1.05]	** < 0.001**	n/i	n/i
Initial presentation mode at site: hospital admission	3.1 [2.13–4.61]	** < 0.001**	3.2 [1.71–5.99]	** < 0.001**
SSA(Ro) antibodies	0.68 [0.48–0.97]	**0.03**	n/i	n/i
SSB(La) antibodies	0.34 [0.21–0.57]	** < 0.001**	n/i	n/i
Rheumatoid factor	0.49 [0.32–0.76]	**0.001**	n/i	n/i
Allergy affected patients	0.68 [0.48–0.98]	**0.04**	n/i	n/i
Drug-related allergy	0.64 [0.42–0.99]	**0.04**	n/i	n/i
Seasonal allergy	0.61 [0.38–0.99]	0.05	n/i	n/i
IgG	0.93 [0.88–0.99]	**0.02**	0.92 [0.86–0.998]	**0.04**
WBC	1.15 [1.02–1.29]	**0.02**	n/i	n/i
Neutrophils, absolute	1.11 [0.97–1.28]	0.1	n/i	n/i
Eosinophils, absolute	1.5 [1.23–1.94]	**0.001**	1.33 [1.05–1.68]	**0.02**
Eosinophils, relative	1.002 [1.001–1.004]	**0.01**	n/i	n/i
CK	1.003 [1.0005–1.005]	**0.02**	n/i	n/i

*OR* odds ratio, *n/i* not included into multivariate regression model, *WBC* white blood cells, *CK* Creatine kinase, *CI* confidence interval, bold values refers statisitcally significant finding

## Discussion

Our data suggest that patients with neurological involvement of Sjögren’s syndrome have a different clinical phenotype compared to patients with Sjögren’s syndrome but without neurologic involvement. This hypothesis has been proposed in previous works [8, 11, 20] since awareness and understanding of neurological involvement in Sjögren’s syndrome has increased in recent years [11, 26]. Nevertheless, this is the first time that this has been further supported in a cohort of comparable size.

### Large proportion of patients with neurological involvement in primary Sjögren’s syndrome

It is striking that the analyzed cohort of primary Sjögren’s syndrome includes a large proportion (46%) of patients with additional neurological involvement, although neurological involvement was assumed only for strictly selected neurological symptoms. This high rate of neurological involvement in Sjögren’s syndrome might be explained by an increased awareness of this entity at our university hospital due to many completed and ongoing research projects combined with a clinical specialization in patients with Neuro-Sjögren. The high frequency of neurological involvement is further explained by the intensified clinical screening that has been integrated into the routine diagnostic algorithm of both the Department of Neurology and the Department for Rheumatology and Immunology at our site in recent years. The thorough workup of this cohort is also reflected in the high number of salivary gland biopsies performed. Our data support the hypothesis that neurological involvement in Sjögren’s syndrome has been previously underestimated and underdiagnosed.

### Distinct clinical phenotype

Our analysis suggests several differences regarding the clinical phenotype of neurologic involvement of Sjögren’s syndrome compared to Sjögren’s syndrome without neurological involvement. First, patients with pSSN showed a more balanced female to male ratio (65% vs. 85% female). This tendency has been described before in smaller cohorts and cohorts with mixed primary and secondary Sjögren’s syndrome and neurological involvement [11–13, 27]—but explanations remain notional. Second, symptom onset was found to be at a significantly higher age in patients with additional neurological involvement (median 55 [IQR 44–65] vs. 45 [IQR 33–56] years). This finding is consistent with a previously published analysis by the Sjögren Big Data Consortium describing an increasing frequency of peripheral nervous system involvement with older age for patients with Sjögren’s syndrome [28]. Third, patients with pSSN were initially more likely to be hospitalized, which may be an indicator of a more severe disease course. The higher hospitalization rate in patients with Sjögren’s syndrome and neurological disorders was also found in the nationwide retrospective analysis using the French Health insurance database 2022 [29]. And lastly, some laboratory findings differed between the two subgroups: Notably, patients with Sjögrens syndrome and neurological involvement lacked rheumatoid factor and SSA(Ro)/SSB(La) antibodies in the subgroup comparison. This is an interesting point, as previously published data indicate a distinct phenotype in patients with Sjögren’s syndrome and older age [28]. Data of the respective investigation suggested that older age of patients with Sjögren’s syndrome was associated with a higher frequency of oral dryness and a lower frequency of all autoantibodies and immunological markers except for cryo-globulins. Another study by Veenbergen at. al. also found that autoimmune antibodies were more common in younger patients with Sjögren’s syndrome [30]. Furthermore, our analysis found higher CK values in patients with pSSN, most likely reflecting progressive primary (i.e., myositis) or secondary (i.e., motor neuropathy) muscular damage. There were even two independent laboratory predictors of neurologic involvement in Sjögren’s syndrome: slightly lower absolute values for IgG and slightly higher absolute values of eosinophils—albeit still within the reference range.

### Neurological features

Interestingly, several neurological disorders showed a striking clustering in the entire cohort without being established as neurological manifestations of Sjögren’s syndrome. For example, 6 patients (1%) were additionally diagnosed with myasthenia gravis, based on suggestive electro-diagnostic findings on repetitive stimulation and/or myasthenia-associated antibodies. The association of myasthenia gravis and Sjögren’s syndrome has been discussed in several case reports although it has not yet been investigated on a larger scale [31]. Another notable accumulation was found for multiple sclerosis (5%) and NMOSD (1%). Both entities have been described as associated with Sjögren’s syndrome [13, 32–36], but a direct pathophysiological link has not yet been established. Most importantly, 4% of our cohort reported a history of stroke and another 2% reported a history of TIA. This finding is consistent with previous analyses showing a particular cardiovascular risk for patients with Sjögren’s syndrome [37–40]. Hypothetically, increased cardiovascular risk could also cause neuropathy via destruction of the vasa nervorum, as is the case in other diseases such as diabetes [41]. It remains speculative whether the increased incidence of cardiovascular events in Sjögren’s syndrome is due to a primary disease-associated patho-mechanism or secondary to lifestyle factors (i.e., smoking, medication, mobility, pain).

The diagnostic algorithm should therefore be thoroughly pursued in patients presenting with neurological features, especially in cases of neuropathy, myasthenia, multiple sclerosis, NMOSD, and cerebrovascular incidents.

### ESSDAI—NISSDAI

Patients with neurological involvement had significantly higher ESSDAI values than patients with Sjögren’s syndrome but without neurological manifestations (*p* < 0.0001). This effect is a natural consequence of the included neurological background sub-scores (PNS, CNS, muscular). As discussed above, many problems arose with the ESSDAI assessment when evaluating neurological patients in a cohort of this size. Therefore, the NISSDAI was developed as an initial proposal for a more adaptive scoring system for neurological symptoms in Sjögren’s syndrome. Further studies are needed to validate this scoring system.

### Limitations

Interpretation of the data is limited due to the retrospective design of our study. Further prospective studies are needed to validate the observed findings.

## Conclusions

Patients with Sjögren’s syndrome and neurologic involvement in this study had a different clinical phenotype than patients with Sjögren’s syndrome without neurologic involvement. They also constituted a large proportion of the analyzed cohort. Therefore, our data support the hypothesis that neurological involvement in Sjögren’s syndrome has been underestimated until now. We further deduce that intensified screening for neurologic involvement should be included in the diagnostic algorithm for Sjögren’s syndrome, especially in male patients of older age and severe disease course requiring hospitalization.

## Data Availability

All data are available upon reasonable request.
